# Lamin A/C as a Molecular Link Between Nuclear Organization, Chromatin Dynamics, and Tumor Progression

**DOI:** 10.3390/cells15060501

**Published:** 2026-03-11

**Authors:** Cecilia Foglini, Katia Scotlandi, Michela Pasello

**Affiliations:** Laboratory of Oncology Research and Functional Genomics (ONCOGEN), IRCCS Istituto Ortopedico Rizzoli, 40136 Bologna, Italy; cecilia.foglini@ior.it

**Keywords:** lamin A/C, nuclear mechanics, chromatin organization, epigenetic regulation, nuclear lamina, cellular plasticity, mechanotransduction, cancer progression, tumor heterogeneity

## Abstract

**Highlights:**

**What are the main findings?**

- Lamin A/C is a dynamic mechano-epigenetic regulator that links nuclear mechanics with chromatin organization and transcription.
- In cancer, lamin A/C functions in a strongly context-dependent manner, yielding heterogeneous and sometimes opposing prognostic associations across tumor types.

**What are the implications of the main findings?**

- Lamin A/C might be viewed as a nuclear rheostat that regulates tumor cell behavior along a spectrum from deformability and plasticity to mechanical robustness and genome stability.
- Clinical translation requires context-dependent strategies that use lamin A/C features for patient stratification and target its associated pathways for treatment while preserving normal tissue function.

**Abstract:**

Lamin A/C is emerging as a promising candidate regulator at the intersection of nuclear mechanics, chromatin organization, and gene regulation, linking structure and regulation, mechanics and epigenetics, constraint and plasticity. Lamin A/C was previously considered a static structural scaffold; however, it is now recognized as a dynamic component of nuclear organization that links physical cues to epigenetic and transcriptional states. Lamin A/C regulates three-dimensional genome structure, constrains chromatin mobility, and influences cell transitions between plastic and committed states through its interactions with heterochromatin at the nuclear periphery and active chromatin domains in the nuclear interior. In cancer, these functions appear to be dependent on the context. Lamin A/C has been implicated in crucial biological processes, including invasion, survival under mechanical stress, lineage plasticity, and therapeutic response. Its prognostic value varies across tumor types. This heterogeneity indicates that lamin A/C does not function as a traditional oncogene or oncosuppressor; instead, it operates as a nuclear rheostat, influencing the behavior and development of tumor cells. This review examines the potential clinical benefits of lamin A/C while considering its implications for normal tissue functions. It aims to improve understanding of cellular adaptability and vulnerability in cancer through the exploration of lamin A/C biology.

## 1. Introduction

The eukaryotic nucleus is both a repository of genetic information and a highly organized, mechanically responsive organelle in which spatial architecture is tightly linked to the genome function. A key factor in nuclear organization is the nuclear lamina, a filamentous protein network underlying the inner nuclear membrane (INM) that provides structural support while actively regulating chromatin organization and gene expression. The lamina is composed of type V intermediate filaments, including A-type lamins (lamin A and lamin C) and B-type lamins (lamin B1 and B2). Among these, lamin A/C is distinctive in functioning not only as a structural scaffold but also as a dynamic integrator of mechanical, genomic, and transcriptional cues, positioning it as a mechano-epigenetic regulator at the interface of nuclear architecture, cell fate, and disease. In this review, we synthesize current knowledge of lamin A/C biology with a focus on cancer. We first outline mechanisms regulating lamin A/C expression, maturation, and post-translational modification. We then discuss how lamin A/C organizes chromatin at the nuclear periphery and within the nucleoplasm, integrates mechanical and epigenetic cues during differentiation, and contributes to tumor progression, metastasis, and therapy response. Finally, we propose a mechano-epigenetic framework to reconcile seemingly conflicting findings and guide future diagnostic and therapeutic approaches. Here, we refer to nuclear rheostat in an operational sense: stepwise changes in lamin A/C activity (shaped by abundance, isoform balance, maturation, and post-translational state) produce dose-dependent or thresholded effects on nuclear mechanics and chromatin organization. A key feature is partial reversibility, such that restoring lamin A/C toward baseline reverses, at least in part, the associated mechanical and transcriptional outputs. This framework helps explain why different tumors adopt distinct lamin A/C operating states depending on whether progression favors deformability, mechanical robustness, therapy resistance, or plasticity.

## 2. Mechanisms Regulating Expression, Maturation, and Post-Translational Modification of Lamin A/C

### 2.1. LMNA Gene, Lamin A/C Isoforms, and Filament Architecture

**Figure 1:**
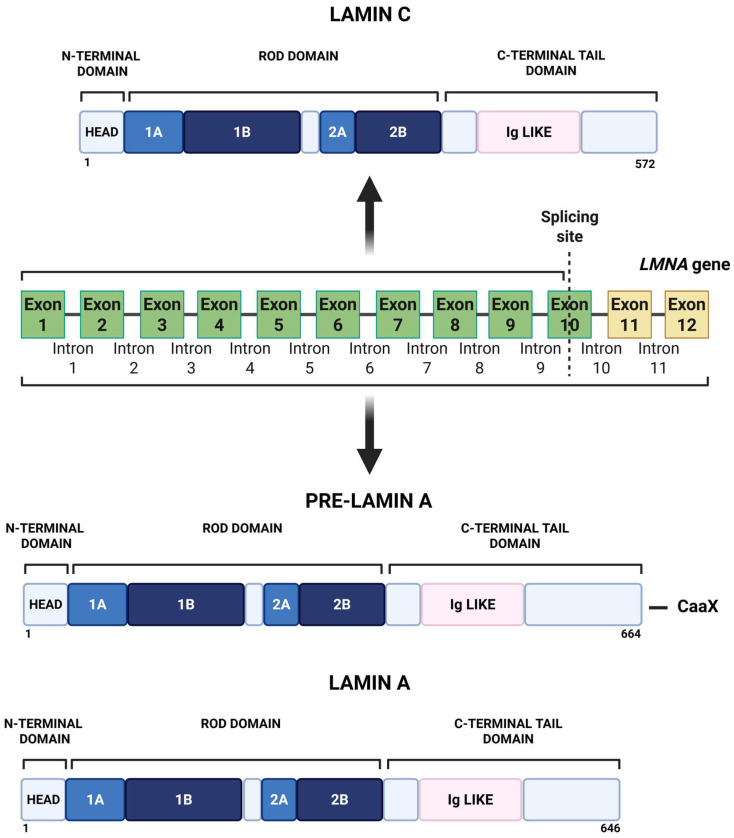
Schematic representation of the *LMNA* gene and the structural organization of lamin C, prelamin A, and mature lamin A. The *LMNA* gene consists of 12 exons. Alternative splicing at exon 10 generates lamin C. Inclusion of exons 11 and 12 produces prelamin A, a precursor protein containing a C-terminal -CaaX motif that undergoes post-translational processing to generate mature lamin A. All lamin isoforms share a conserved N-terminal head domain and a central rod domain (subdomains 1A, 1B, 2A, and 2B), followed by a C-terminal tail domain containing an immunoglobulin (Ig)-like fold. Protein lengths (in amino acids) are indicated. Created in BioRender. Pasello, M. (2026) https://BioRender.com/2xrj9x1 (accessed on 2 March 2026).

Lamin A and lamin C are A-type lamins, commonly referred to collectively as lamin A/C, that are produced from the same gene, *LMNA*, through alternative splicing [1]. Lamin A is synthesized as a precursor, prelamin A, which contains a C-terminal CAAX motif, whereas lamin C lacks this motif and is produced as a shorter polypeptide. Despite these differences, both isoforms share the canonical tripartite architecture of intermediate filaments: a small globular N-terminal head, a central α-helical rod domain, and a C-terminal tail containing a nuclear localization signal and an immunoglobulin (Ig)-like fold. A schematic representation is shown in Figure 1.

The central rod domain comprises four coiled-coil segments that mediate parallel dimer formation. These dimers assemble longitudinally in a head-to-tail orientation to form protofilaments, which subsequently associate laterally through antiparallel, staggered interactions to generate higher order lamin filaments [2]. Recent structural studies have improved this model, revealing multiple longitudinal interaction modes (often termed A11 and A22) and extensive combinations of N- and C-termini in head-to-tail contacts [3]. Cryo-electron tomography further indicates that lamin filaments are remarkably thin (~3.5 nm in diameter) and exhibit shorter tail-to-tail spacings than previously predicted [4]. This polymer architecture helps explaining the mechanical flexibility of lamins compared to cytoplasmic intermediate filaments and provides the scaffold upon which processing events and post-translational modifications act to fine-tune lamin assembly, localization, and interactions with membranes and chromatin.

### 2.2. Physiological Processing and Post-Translational Modifications of Lamin A/C

**Figure 2:**
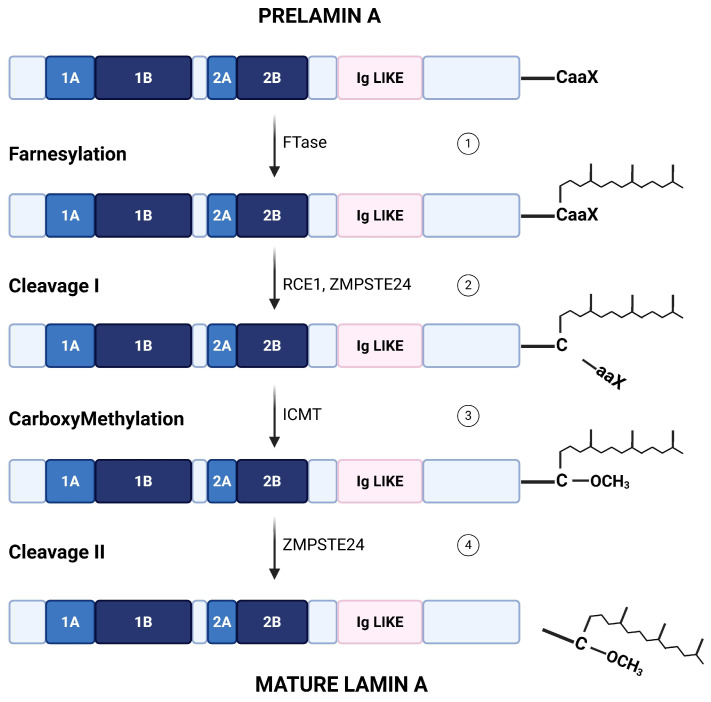
Post-translational processing of prelamin A into mature lamin A. Prelamin A is synthesized with a C-terminal CaaX motif and undergoes a series of sequential post-translational modifications: (1) the C-terminal cysteine of the CaaX motif is farnesylated by FTase; (2) the -aaX is removed by the action of RCE1 and ZMPSTE24; and (3) the newly exposed C-terminal farnesylcysteine is methylated by ICMT; (4) the C-terminal region, including the farnesylcysteine methyl ester, is clipped off by ZMPSTE24 and degraded, generating mature lamin A. Functional domains of lamin A, including the head (1A, 1B), rod (2A, 2B), and immunoglobulin-like (Ig-like) domains, are indicated. Created in BioRender. Pasello, M. (2026) https://BioRender.com/tagymyk (accessed on 28 January 2026).

At the most fundamental level, lamin A biogenesis is governed by a defined post-synthetic processing pathway. Prelamin A undergoes a series of sequential enzymatic steps: farnesylation of the CAAX cysteine by farnesyltransferase, proteolytic cleavage of the -AAX tripeptide by ZMPSTE24 (or the related CAAX protease RCE1), an endoplasmic reticulum (ER)/inner nuclear membrane zinc metalloprotease, carboxyl methylation by ER methyltransferase ICMT and final ZMPSTE24-mediated removal of the farnesylated tail to yield mature lamin A [5,6]. As a result, mature lamin A lacks the farnesyl group (a schematic representation is shown in Figure 2). Inactivation of this maturation pathway by mutations in *LMNA* or *ZMPSTE24* genes leads to the accumulation of permanently farnesylated prelamin A species, such as progerin. These aberrant forms cause nuclear deformation, DNA damage, and the development of premature aging laminopathies, including Hutchinson–Gilford progeria syndrome and restrictive dermopathy [7,8,9]. In contrast to lamin A, lamin C bypasses this processing cascade entirely, as it does not contain a CAAX motif [10].

In addition, lamins are subject to a highly coordinated network of post-translational modifications, including phosphorylation, acetylation, SUMOylation, methylation, ubiquitination, and O-GlcNAcylation. All these modifications regulate lamin solubility, assembly state, and interactions with chromatin and nuclear envelope proteins [11,12,13,14].

While several post-translational modifications (PTMs) primarily have physiological roles in lamin dynamics and proteostasis, a subset is recognized as being repurposed by oncogenic signaling and stress pathways, thereby linking lamin state to cancer-relevant phenotypes discussed later (see Section 4).

Phosphorylation is the most well-known and dynamic post-translational modification of lamin A/C. More than 70 phosphorylation sites have been identified, mostly within the head and tail domains [15,16]. Kinases such as cyclin-dependent kinase 1 (CDK1), protein kinase C (PKC), protein kinase B (AKT), mitogen-activated protein kinases (MAPKs), and protein kinase A (PKA) target these residues to regulate lamin solubility, filament disassembly, and chromatin association [17,18,19]. Several studies demonstrated that CDK1-mediated phosphorylation of Ser22 and Ser392 is essential for nuclear lamina disassembly during mitotic entry [20,21]. Recent research has shown that coordinated CDK1-dependent phosphorylation at N- and C-terminal residues (Thr19/Ser22 and Ser390/Ser392) is required for proper lamina dynamics. Specifically, phospho-mimetic 4D mutants (T19D/S22D/S390D/S392D) destabilize lamin filaments during interphase, whereas non-phosphorylatable 4A mutants (T19A/S22A/S390A/S392A) block depolymerization and, when engineered into the endogenous *LMNA* locus, lead to nuclear abnormalities and micronuclei formation during telophase. These findings highlight the importance of tightly regulated phosphorylation and dephosphorylation cycles for nuclear envelope plasticity and genomic stability [22]. Additional phosphorylation sites, such as Tyr45, Ser404 and Thr424 further contribute to nuclear reorganization and mechanoadaptation during interphase [23,24]. In the context of cancer, it is noteworthy that interphase Ser22 phosphorylation has emerged as a marker of more soluble nucleoplasmic lamin pools, providing a mechanistic explanation on how growth factor signaling, such as AKT activity, may soften nuclei, facilitate migration through confined environments and link mechanical adaptation to transcriptional reprogramming.

Acetylation is another critical regulatory layer. Major acetylation sites within the coiled-coil rod and Ig-fold domains (e.g., K97, K311, K417, K450, and K470) are catalyzed by the lysine acetyltransferase MOF. Loss of MOF or its cofactors results in lamin A/C hypoacetylation, increased Ser392 phosphorylation, nuclear fragility, and impaired chromatin organization, linking the acetylation status to the stability of lamina and DNA repair capacity [25,26]. This is of relevance in the context of malignancy, given that nuclear fragility and altered DNA damage response can impact sensitivity to genotoxic stress and therapy (see Section 4).

SUMOylation affects lamin localization, turnover, and cellular stress responses. Lamin A interacts with the E2 enzyme UBC9 and is SUMOylated at conserved lysine residues, such as K201, primarily by SUMO2/3 [27,28]. In cell models, including HeLa cells and U2OS cells, and in mouse embryonic fibroblasts (MEFs), lamin SUMOylation is proven to be particularly dominant during mitotic lamina remodeling and has been implicated in nucleophagy [29]. Mutations affecting SUMOylation sites or perturbing SUMO1 localization further highlight the contribution of this modification to nuclear homeostasis [27,30].

Additional post-translational modifications add further regulatory complexity, influencing membrane affinity, lineage programs, and protein turnover. For example, carboxyl methylation of the CAAX cysteine increases hydrophobicity and thereby enhances membrane association of newly processed prelamin A [31,32]. Beyond these CAAX-linked steps, arginine methylation by PRMTs contributes to linking lamin A/C to myogenic transcriptional programs and muscle differentiation [33]. Lamin A/C abundance is actively regulated by the ubiquitin–proteasome pathways, with E3 ligases, including RNF123, HECW2, and Smurf2, contributing to controlled turnover and proteostasis [34,35]. Finally, O-GlcNAcylation within lamin A residues 601–645, a region missing in the progerin variant, has been reported to stabilize lamin A and has been proposed as a molecular link between lamin regulation and nuclear aging phenotypes [13].

These physiological events and post-translational modifications define a regulatory system that coordinates lamin filament assembly, localization, and chromatin engagement. Perturbations of this system can alter nuclear architecture, chromatin organization, and cellular responses to mechanical and genotoxic stress, thereby providing a biochemical basis that may be altered during tumorigenesis.

## 3. Lamin A/C: Role in Structural Stability, Genomic Organization and Cell Fate

Lamin A/C plays many important roles in the cell, including preserving nuclear structural stability and proper nuclear morphology under mechanical stress [36]. The nuclei of *lmna*−/− mouse fibroblasts tend to be more fragile and deform easily under mechanical stress [37], while mutations in lamin A or lamin C can cause lamina instability as well as loss of internal nuclear lamin organization and are associated with various inherited diseases, such as Emery–Dreifuss muscular dystrophy or Dunnigan-type familial partial lipodystrophy [38]. Decreased stability of lamin mutants can lead to altered organization of intranuclear chromatin and loss of gene regulatory functions. Indeed, lamin A/C interacts with nuclear membrane proteins, transcription factors, chromatin regulators, signaling molecules, splicing factors, and chromatin itself to form a nuclear subcompartment involved in maintaining nuclear integrity, nuclear positioning, mitosis, DNA repair, DNA replication, splicing, mechanotransduction and mechanosensing, transcriptional regulation, and genome organization.

### 3.1. Lamina-Associated Domains and the Nuclear Periphery

The genome is hierarchically organized in both space and function: heterochromatin preferentially localizes to the nuclear periphery, whereas euchromatin is enriched in the nuclear interior and near nuclear pores. Specialized contact sites at the nuclear envelope actively maintain this radial organization. Here, the meshwork of lamin filaments is anchored to the inner nuclear membrane (INM) through interactions with INM proteins and the nuclear pore complexes (NPCs) [39]. Lamins cooperate specifically with INM transmembrane proteins, such as lamin-B receptor (LBR), lamina-associated polypeptide 2β (LAP2β), emerin, and LEM-domain proteins (LEMD2, MAN1), to tether repressive chromatin and support the establishment of lamina-associated domains (LADs) [40,41,42]. Different LEM domain proteins mediate heterochromatin binding to lamin A/C depending on cell type and developmental stage. Simultaneously, LAP2β interacts with histones, barrier-to-autointegration factor (BAF), and histone deacetylase 3 (HDAC3), thereby fortifying repressive chromatin environments at the nuclear periphery [43,44,45]. In contrast, NPC proteins are generally associated with transcriptionally active chromatin and contribute to nuclear compartmentalization [41,42]. The developmental stage is a critical factor in determining which nuclear-envelope tether predominates. Solovei et al. [45] demonstrated that peripheral heterochromatin is anchored by LBR in proliferating/early differentiating cells, whereas tethering dependent on lamin A/C takes place during terminal differentiation. This finding indicates a sequential handover in nuclear organization. The loss of both tethers has been shown to result in the dissolution of peripheral heterochromatin, which can lead to an inverted nuclear architecture, suggesting that the radial genome organization is actively maintained.

Genomic mapping approaches (DamID, ChIP-seq), and imaging techniques (FISH, live-cell imaging), have been instrumental to define LADs as large genomic regions (~0.1–10 Mb) characterized by low gene density, late replication timing, and enriched for repressive histone modifications such as H3K9me2/3 and H3K27me3 [46,47]. LADs are dynamically regulated during cell state transitions. Recent studies of biophysical modeling and high-resolution microscopy further indicate that interactions between chromatin and lamina, along with histone methylation and acetylation, play a mechanistic role in shaping heterochromatin morphology both at the nuclear periphery and inside the nucleus [48]. These findings define the nuclear lamina as a central scaffold for chromatin compartmentalization, in which A-type and B-type lamins cooperate to establish and maintain LADs. Disruption of these peripheral anchoring mechanisms can redistribute genomic regions between repressive and permissive compartments, facilitating the activation of transcriptional programs linked to proliferation, motility, or stress responses, an effect that may facilitate oncogenic deregulation. This peripheral scaffold is complemented by more local, sequence-specific lamin–chromatin interactions, which are discussed in the next section.

### 3.2. Direct and Indirect Interactions of Lamin A/C with Chromatin

The property of lamins to bind chromatin directly was first demonstrated in vitro, where purified lamins were shown to associate with native chromatin and core histones [49,50]. Later biochemical studies found that the C-terminus of A-type lamins plays a key role. The Ig-like domain and the area around the nuclear localization signal are responsible for direct DNA interactions, probably through electrostatic complementarity [51,52]. Dimerization of lamin A/C further stabilizes these interactions, enabling the formation of complexes that anchor chromatin at the nuclear lamina. Cryo-electron tomography and structural modeling have provided near-atomic insight into these interfaces. The flexible C-terminal tail of lamin A can extend to nucleosomes positioned approximately 30–50 nm from the lamina and interact with histone surfaces through conserved motifs within the tail region [53]. Deletions in this region accelerate lamin A turnover and reduce its retention at the nuclear envelope, indicating that direct nucleosome binding contributes not only to heterochromatin organization but also to lamina stability itself [54].

Beyond direct binding, lamins organize chromatin through multiprotein complexes at the nuclear envelope. Proximity labeling and proteomic analyses have identified more than 400 nuclear envelope proteins, of which approximately 100 interact directly or indirectly with lamin A/C [55,56]. Central to this network are LEM-domain proteins such as LAP2, emerin, and MAN1, which bind both lamin A/C and BAF, a DNA-bridging protein capable of linking two double-stranded DNA molecules [57]. The lamin A/C-BAF-LEM complex provides several DNA-binding interfaces that strengthen heterochromatin anchoring at the nuclear periphery, along with direct lamin–nucleosome contacts. In parallel, LBR, which interacts with lamin B and CBX5/HP1, tethers H3K9me3-enriched chromatin to the lamina; combined loss of LBR and lamin A results in near-complete disorganization of peripheral heterochromatin [45], underscoring their complementary roles. Histone modifications are tightly integrated into these interactions. Lamin A associates with the histone methyltransferase Suv39h1, thereby supporting the stability of the H3K9me3 mark; *LMNA* knockout reduces H3K9me3 levels and increases chromatin plasticity [58,59]. B-type lamins recruit the Polycomb repressive complex (PRC2) to sustain H3K27me3 distribution, and depletion of lamin B1 leads to loss of this mark, altered replication time, and reorganization of repressive chromatin domains [60,61].

Together, these studies suggest that lamin–chromatin interactions operate on multiple levels: direct nucleosome binding, indirect bridging through LEM-domain and other inner nuclear membrane proteins, and modulation of the histone modification landscape. These interactions provide both mechanical stability and functional adaptability to the nucleus. Therefore, oncogenic perturbations at any of these nodes, from Suv39h1 or PRC2 recruitment to the stability of LEM/BAF interactions, could shift the balance between repressed and active chromatin domains, leading cells to aberrant transcriptional programs.

Beyond lamin–chromatin interactions occurring at the nuclear periphery, a distinct nucleoplasmic pool of lamin A/C is present within the nuclear interior [62]. This more soluble fraction contributes to euchromatin organization and participates in the regulation of active chromatin by constraining locus mobility and modulating chromatin accessibility [59,63]. Within the nuclear interior, lamin A associates with the LAP2α isoform, which binds lamin A but not lamin C [64]. This interaction defines a class of euchromatic lamin-associated domains, sometimes referred to as A-LADs, that are distinct from the classical heterochromatic LADs at the nuclear periphery: while LAD-associated lamin A/C typically engages megabase-scale heterochromatin and contributes to stable tethering at the nuclear lamina, nucleoplasmic lamin contacts occur at finer genomic scales and are enriched within more permissive chromatin contexts [65]. Depletion of LAP2α abolishes nucleoplasmic lamin A, redistributing it to the nuclear envelope and reinforcing its association with repressive domains, thereby revealing a dynamic balance between peripheral repression and nucleoplasmic regulation.

A defining feature of the nucleoplasmic pool is its phosphorylation state. Phosphorylation at Ser22 and Ser392, which is generally associated with lamina disassembly during mitosis, also occurs during interphase and marks more soluble, dynamic forms of A-type lamins [66,67]. Phosphorylation of lamin A at Ser22 associates with active enhancers outside classical LADs, linking lamins to transcriptionally suited chromatin domains [68]. In this context, phosphorylation acts as a molecular switch that turns lamin A from a stable structural component of the lamina into a transient regulator of active chromatin. Consistently, LAP2α-dependent nucleoplasmic lamin A/C is a highly mobile, low-assembly pool required for chromatin regulation at both genome-wide and locus-specific scales [69]. Importantly, the extent and genomic distribution of lamin–enhancer association is likely context-dependent, reflecting the active enhancer landscape of a given cell type and cellular state, as well as regulators of nucleoplasmic lamin availability and signaling inputs that change phosphorylation states and residence time at regulatory regions, thus influencing the activation of genes involved in cell proliferation, survival or mechanoadaptation.

The dual localization of lamin A/C anchoring heterochromatin within peripheral LADs while engaging active regions in the nucleoplasm positions the molecule as a central determinant of three-dimensional genome organization. Through continually balancing interactions between repressive and permissive domains, lamin A/C contributes to nuclear compartmentalization, modulates gene expression variability, and couples nuclear structure to transcriptional plasticity during differentiation and stress adaptation.

### 3.3. Regulation of Cell Fate

Lamin A/C is induced as embryonic stem cells (ESCs) differentiate and contributes to stabilization of chromatin organization during lineage commitment [70]. In undifferentiated ESCs, chromatin is highly mobile and permissive, characterized by high histone acetylation and low levels of repressive marks such as H3K9 methylation, which support transcriptional flexibility and pluripotency [71,72]. During early differentiation, this epigenetic landscape progressively stabilizes as acetylation decreases and repressive modifications accumulate. Ectopic *LMNA* expression in wild-type ESCs restricts heterochromatin dynamics, likely through direct interactions with core histones and through tethering chromatin to the nuclear lamina [50,73], thereby functionally mimicking differentiation. *LMNA* expression activates epigenetic pathways that repress pluripotency: increased H3K9 methylation catalyzed by G9a cooperates with lamin A/C to limit chromatin plasticity, whereas persistent hyperacetylation maintains an open chromatin configuration compatible with pluripotency and facilitates somatic cell reprogramming [59,74]. By establishing stable lamina–chromatin interactions, lamin A/C not only reduces heterochromatin mobility but may also suppress pervasive transcription by sequestering genomic regions at the nuclear periphery. Accordingly, in pluripotent cells the absence of lamin A/C supports a highly dynamic, acetylation-rich chromatin state, whereas its acquisition during differentiation stabilizes genome organization and couples with nuclear architecture to epigenetic repression of pluripotency programs [75,76,77].

There is strong evidence of lamin A/C involvement in cell differentiation, in particular in adipogenic, osteogenic, myogenic, and cardiogenic directions (for a review, please refer to Malashicheva et al. 2021 [78]). Lamin A/C preserves accurate lineage specification during early development. As an example, Wang Y. et al. [79] showed a key role of lamin A/C in keeping cardiomyocyte lineage-specific genes in pluripotent stem cells silent, thus preventing aberrant cardiovascular cell fate choices during development. In contrast, loss of *LMNA* in ESCs disrupts chromatin compaction and the spatial organization of cardiac gene loci, leading to premature cardiomyocyte differentiation, cell cycle withdrawal, and abnormal contractility. This imbalance depends on the cardiac regulator Gata4: *LMNA* depletion activates Gata4, a transcriptional factor that is specifically associated with lamin A/C within LADs [80], suggesting that modulation of lamin A/C levels shifts cells from a fluid, permissive chromatin landscape to a more constrained, lineage-stabilized state.

In human adipose-derived stem cells (ASCs), *LMNA* has been observed to associate with the promoters of more than a quarter of expressed genes. This association involves the contact of spatially restricted sub-promoter regions, both within and outside lamin-enriched domains [81]. These interactions modulate local transcription in a chromatin-context-dependent manner, thereby linking *LMNA* occupancy to histone modification patterns. In undifferentiated ASCs, lamin A/C binding at promoters, at upstream or near transcription start sites, correlates with transcriptional inactivity. When *LMNA* is downregulated, lamin separates from these promoters, remodeling repressive and permissive histone marks and increasing transcriptional permissiveness, although this process alone is not sufficient for full activation. During adipogenic differentiation, promoters of adipogenic genes lose lamin A/C association as they become transcriptionally active, indicating that detachment from lamin A/C is a precondition for strong induction of lineage-specific programs [81]. Genome-wide mapping revealed that only about one-third of lamin-bound loci reside at the nuclear periphery, whereas the majority occupy transient positions inside the nucleus [47], supporting a model of flexible, developmentally regulated lamin–chromatin interactions. Lamin A/C has been observed to disengage from promoters of genes that activate the program, still maintaining its binding to pluripotency or non-lineage genes [82,83], thereby contributing to transcriptional repression during lineage restriction. Importantly, high lamin A/C enrichment within a genomic neighborhood correlates with repression of directly bound genes rather than nearby unbound loci, indicating that lamin A/C primarily acts locally rather than as a purely radial “shell” determinant [81].

**Figure 3:**
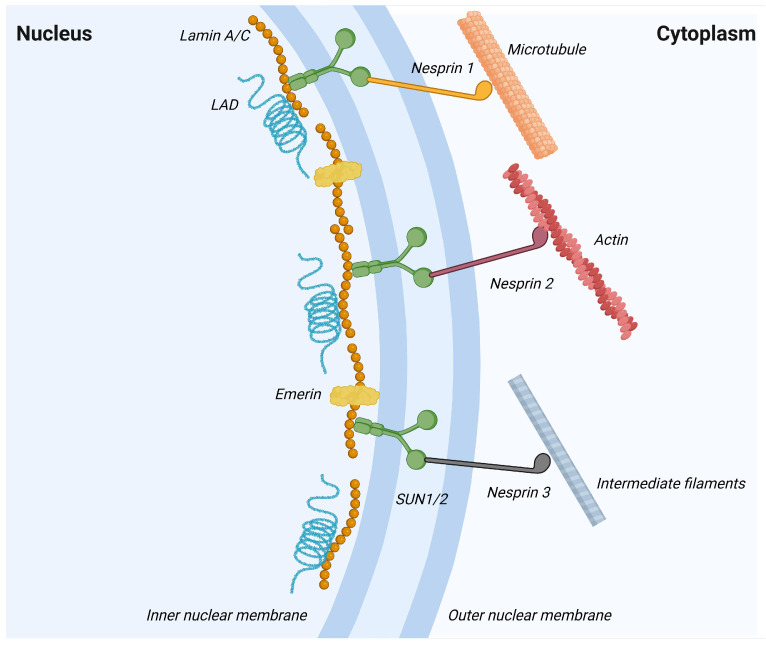
Schematic representation of the linker of nucleoskeleton and cytoskeleton (LINC) complex spanning the nuclear envelope. SUN1/2 proteins are embedded in the inner nuclear membrane, where they interact with nuclear lamins (lamin A/C) and lamina-associated domains (LADs), as well as inner nuclear membrane proteins such as emerin. In the perinuclear space, SUN proteins bind to nesprins. Nesprin-1 connects the nucleus to microtubules, nesprin-2 to actin filaments, and nesprin-3 to intermediate filaments, thereby mechanically coupling the nucleoskeleton to different cytoskeletal networks in the cytoplasm. Created in BioRender. Pasello, M. (2026) https://BioRender.com/i7dzyee (accessed on 28 January 2026).

Lamin A/C also participates in mechanosignaling, a process considered essential for regulating cell differentiation [84,85]. Mechanosignaling refers to the conversion of extracellular mechanical cues from the extracellular matrix (ECM) into intracellular biochemical and transcriptional responses, transmitted through the cytoskeleton and, via the linker of the nucleoskeleton and cytoskeleton (LINC) complex, to nuclear lamins (see Figure 3).

Although the precise molecular mechanisms affecting stem cell fate are still poorly understood, it is believed that during differentiation lamin A/C integrates information from the microenvironment and rearranges chromatin structures, resulting in activation or repression of differentiation-related genes [23,86]. Substrate stiffness is a key regulator of multipotent mesenchymal stem cell (MSC) differentiation: stiff matrices increase lamin A/C expression and reduce its phosphorylation, stabilizing the lamina and promoting osteogenesis, whereas soft matrices lower lamin A/C levels and favor adipogenesis [87,88]. Recent research reports that culturing myoblasts and fibroblasts on extremely soft substrates with stiffness of 0.2 kPa and by knocking out *LMNA* increases nuclear deformability and induces neural-related gene expression, biasing cells toward neural-like differentiation. CUT&Tag and chromatin analyses have linked lamin A/C loss to disruption of lamin B1-associated domains and redistribution of H3K9me2/3-labeled heterochromatin toward the nucleoplasm, connecting mechanical softening to chromatin decompaction and transcriptional reprogramming; conversely, stiff environments maintain high lamin A/C levels and favor myogenic gene expression [89]. On stiff substrates, the retinoic acid receptor RARG binds the *LMNA* promoter, while lamin A/C promotes nuclear accumulation of RARG, forming a circular loop that supports osteogenic commitment [90].

Lamin A/C also regulates actin organization and cytoskeletal tension that affects YAP/TAZ nuclear localization and MKL1/SRF activity; this mechano-responsive axis is robust but not necessarily linear, as changes in matrix rigidity and lamin A/C levels do not translate into a simple one-to-one YAP/TAZ response [90,91]. In parallel, lamin A/C is involved in osteogenic pathways such as Wnt/β-catenin, Notch, and Runx2, supporting osteoblast differentiation [92,93,94,95], while constraining adipogenesis by sequestering PPARγ at the nuclear periphery; when lamin A/C is reduced, PPARγ is released and adipogenic programs are favored [96].

Correct post-synthetic processing of lamin A is also essential for cell lineage decisions. Accumulation of immature prelamin A, for example, upon ZMPSTE24 deficiency or farnesylation inhibition, impairs osteogenic differentiation and can stall adipogenesis, highlighting that both lamin A abundance and its maturation state influence mesenchymal fate balance [97,98]. In other words, the same biochemical processing steps that define “mature” lamin A also function as a rheostat that tunes how stem and progenitor cells respond to mechanical and transcriptional cues.

Lamin A/C also interacts with signaling pathways involved in cell development and differentiation, such as the Wnt/β-catenin, Notch, TGF-β/Smad, and Mitogen-Activated Protein Kinase (MAPK). For instance, lamin A/C was found to sequester c-Fos, a transcription factor that regulates differentiation, at the periphery of the nucleus, thereby reducing its DNA-binding activity [99]. Interaction of lamin A/C with c-Fos is disrupted due to phosphorylation of c-Fos by MAPK ERK, supporting the involvement of lamin A/C in the regulation of MAPK pathway activity [100].

Taken together, these data define lamin A/C as a central integrator of stem and progenitor cell behavior through two interconnected mechanisms: mechanical coupling of the cytoskeleton and ECM to the nucleus, and modulation of transcriptional networks that control fate decisions. By connecting chromatin organization, nuclear mechanics, and signal transduction, lamin A/C provides a structural and regulatory framework that drives exit from pluripotency and stabilization of lineage-specific identities. A note of caution is warranted, however, because the direction and magnitude of these effects are highly context-dependent across the diverse experimental models used (e.g., pluripotent stem cells, mesenchymal stromal cells, fibroblasts, myoblasts, and lineage-committed progenitors). Nevertheless, perturbations of this framework through altered lamin A/C levels, processing, or mechano-signaling have been largely associated with normal and tumor cell dedifferentiation and plasticity, supporting a key role for this filament in physiology and diseases.

**Figure 4:**
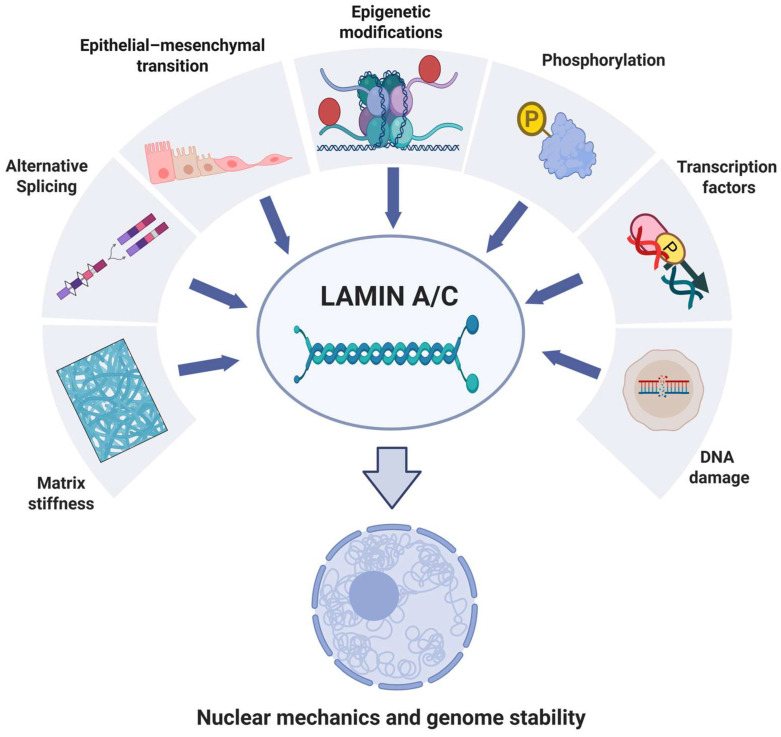
Schematic overview illustrating the multiple regulatory mechanisms that modulate lamin A/C. These include epigenetic modifications, phosphorylation, transcription factor activity, DNA damage responses, alternative splicing, extracellular matrix stiffness and epithelial–mesenchymal transition. These diverse inputs converge on lamin A/C to influence its structure and function within the nuclear lamina. Lamin A/C then plays a central role in controlling nuclear mechanics, chromatin organization and genome stability, thereby linking mechanical and biochemical cues to nuclear integrity and cellular behavior. Created in BioRender. Pasello, M. (2026) https://BioRender.com/b6u4jce (accessed on 28 January 2026).

The schematic representation in Figure 4 recapitulates the aforementioned factors that regulate lamin A/C expression.

## 4. Lamin A/C in Tumors

Due to their diverse functions and numerous interactions, lamin A/C has attracted attention in cancer [101]. Aberrant expression of nuclear lamin A/C and their downstream effects are reported in multiple tumor types, with lamins implicated in abnormal nuclear morphology, disrupted signaling pathways, and genomic instability [102,103,104,105]. Changes in lamin A/C within the nuclear lamina influence cellular stiffness and create a context-dependent balance between cell migratory efficiency (deformability, pore transit, shear sensitivity) and cell survival under mechanical stress (hemodynamic shear, confinement-induced damage). Thus, it comes as no surprise that lamin A/C expression and function are frequently altered in tumors. However, the precise role of lamin A/C levels in cancer remains uncertain, and both increased and decreased levels of lamin A/C correlated to poor prognosis in human cancers (for a review see Dubik et al. 2020, and Chauhan et al. 2025 [101,106]).

### 4.1. Aberrant Mechanisms in the Regulation of Lamin A/C Expression, Processing, and Turnover

*LMNA* mutations, deletions, or copy-number alterations are comparatively rare across most human tumors [107]. In contrast, the expression of lamin A/C is frequently altered. Aberrant regulation of lamin A/C can occur through multiple mechanisms, including alternative splicing of the *LMNA* gene, epigenetic regulation of the gene, post-translational modifications, and abnormal pathways that control its degradation or stabilization. In carcinoma, including breast, colon, liver, lung, ovary, thyroid, and prostate cancers, a variety of studies have described alternative splicing of *LMNA*, which shifts the balance between lamin C and lamin A, as a way to increase the lamin C to lamin A mRNA ratio compared to matched normal tissues [108,109]. Earlier studies in leukemias and lymphoma demonstrated that expression of lamin A/C can be downregulated in the absence of structural gene alterations through epigenetic silencing of *LMNA* via CpG island promoter hypermethylation [110,111]. In addition, a variety of post-translational mechanisms further participate in the regulation of lamin A/C expression and function in malignant cells, in part by repurposing the same enzymatic pathways that govern lamin physiology. Multiple and diverse mechanisms have been described in different tumors and models, making a challenge the systematic detection of lamin A/C alteration in cancer. As an example, in ovarian cancer, overexpression of caspase-6 correlates with increased lamin A cleavage and reduced lamin A levels [112]. Conversely, in non-small cell lung carcinoma, interaction with the intermediate filament protein nestin stabilizes lamin A/C by protecting it from proteasomal degradation [113].

### 4.2. Lamin A/C, Nuclear Mechanics, and Tumor Hallmarks

Lamin A/C is involved in cancer progression through its extensive interaction network. Recent research has brought new insights into the potential of lamin A/C as both a therapeutic target and a molecular biomarker by investigating how its dysregulation disrupts oncogenic pathways and alters protein–protein interactions, which, in turn, affect cellular phenotypes across diverse malignancies.

Dysregulated interactions with nuclear envelope partners such as LAP2α and emerin have been associated with aggressive tumor phenotypes [114]. Components of the LINC complex, including emerin and lamin A/C, are frequently downregulated in tumors, suggesting a system-level contribution to disease development [115,116,117,118]. Emerin interacts with lamin A and contributes to nuclear stiffness; it is reduced in various carcinomas, and its loss is associated with nuclear blebbing and defective nuclei in prostate and breast cancer cell lines. Emerin also marks cells with unstable nuclear morphology in patient tissues and circulating tumor cells [119,120,121]. In lung and thyroid carcinomas, decreased expression of emerin and lamin A/C correlates with undifferentiated histotypes and nuclear atypia [122,123,124]. Similarly, osteosarcoma cell lines display significantly lower levels of A- and B-type lamins and emerin compared with normal osteoblasts, and reduced lamin A levels are associated with increased proliferation and migration [125,126,127].

Nuclear–cytoplasmic connections mediated by the LINC complex further integrate lamin A/C into mechanotransduction pathways relevant in cancer [128,129]. LINC-dependent coupling protects nuclei from deformation and is essential in tissues exposed to mechanical stress. This coupling allows lamin A/C to redistribute between the nucleoplasm and the nuclear periphery in response to external forces, thereby modulating nuclear stiffness and cell polarization [130]. In Ewing sarcoma cellular model, low lamin A levels correlate with enhanced migration and invasiveness, consistent with impaired mechanosignaling. Re-expression of lamin A restores LINC complex components and cytoskeleton-related effectors such as YAP/TAZ, thereby suppressing migration and invasion [131]. The association between decreased expression of lamin A/C and increased cell motility appears to be a shared feature across multiple cancer models. In neuroblastoma, lamin A/C knockdown increases migration [132], likely by softening the nucleus and facilitating transit through confined spaces [133,134]. Comparable effects have been reported in colorectal, prostate, breast, ovarian, and lung cancers, where reduced lamin A/C expression correlates with enhanced migration, epithelial-to-mesenchymal transition (EMT) features, and metastasis [109,135,136,137,138,139]. However, lamin-dependent mechanics can create stage-specific trade-offs during metastasis: reduced lamin A/C may impair survival under fluid shear stress in circulation, thereby limiting metastatic efficiency [133], whereas elevated lamin A/C can increase shear resistance and, in some contexts, support distal metastatic dissemination [36,140].

Beyond its mechanical role, lamin A/C modulates multiple signaling pathways. Lamin A/C interacts with pRb, c-Fos, SREBP1, and MOK2, and participates in p53, MAPK/ERK, WNT, TGF-β, Notch, and NF-κB signaling cascades [132,141].

Multiple oncogenic signaling pathways directly modify lamin A/C to alter nuclear mechanics and genome stability in cancer. For example, AKT2-driven phosphorylation of lamin A during TGF-β-induced EMT promotes nuclear deformation and chromosomal instability [142,143]. Conversely, AKT1-mediated phosphorylation of lamin A/C at Ser22 softens nuclei in colon cancer cells and enhances migration through confined environments [139]. DNA damage signaling further converges with these mechanical effects: inhibition or loss of ATM reduces *LMNA* transcription and lamin A protein levels, generating more deformable nuclei in breast adenocarcinoma and fibrosarcoma models and linking DNA damage response (DDR) status to nuclear stiffness and invasive potential [144]. Defects in prelamin A processing have also been linked to activation of innate immune-like inflammatory programs, such as NF-κB and interferon-response gene signatures, with downstream cytokine/JAK-STAT signaling (e.g., IL6-STAT1/3), possibly leading to a low-grade, chronic inflammatory state that might promote the growth of tumors [145,146].

In lymphoma, leukemia, and neuroblastoma, epigenetic processes such promoter hypermethylation silence *LMNA*; treatment with demethylating drugs can restore lamin A/C expression and decrease invasion and proliferation [110,111,147]. Proteolytic degradation of lamin A/C by caspase 6 has been associated with ovarian cancer [112], while TPX2-driven modulation of lamin A/C phosphorylation affects lamin stability and cellular behavior [148,149]. These regulatory elements provide mechanistic ways through which oncogenic and stress-response pathways converge on lamin A/C, connecting biochemical signaling to nuclear mechanics and downstream transcriptional states.

Growth-factor signaling in tumor cells and inflammatory activation in myeloid cells simultaneously activate AP-1 programs, including c-Fos, providing an interface between microenvironmental cues and lamin-dependent nuclear regulation [150]. Interactions between lamin A/C and c-Fos affect invasion in colon cancer because GDF15 causes c-Fos separation from lamin A/C, triggering AP-1-dependent transcription and promoting the expression of metastatic genes [151,152]. AKT signaling increases nuclear deformation and migratory ability in highly invasive breast cancer cells by downregulating lamin A/C. In aggressive tumors, this phenotype is associated with high AKT activity and low lamin A levels [139]. Therefore, lamin A/C-dependent nuclear mechanics in prostate and other cancers may interact with altered PI3K/AKT pathway regulation, often due to PTEN loss [153,154,155,156].

Notably, lamin A/C regulation extends beyond tumor cells: mechanical and biochemical signals from the tumor microenvironment can reshape lamin organization and function, influencing chromatin tethering and cell state.

Mechanical inputs from the tumor microenvironment also influence lamin A/C function. Increased matrix stiffness and cell spreading can mask an epitope within the lamin A immunoglobulin domain, reducing lamin–chromatin interactions without altering total *LMNA* mRNA or protein levels [23]. Recent work highlights that lamin A/C regulation in cancer extends beyond malignant cells to the tumor microenvironment (TME). In dermal fibroblasts and cancer-associated fibroblasts (CAFs), phosphorylation of lamin A/C at Ser301 has emerged as a critical determinant of CAF activation downstream of androgen receptor (AR) loss. AR directly associates with lamin A/C and recruits it to the PPP1 phosphatase complex. AR depletion disrupts lamin–PPP1 interactions, increases lamin A/C phosphorylation at Ser301, and redistributes lamin within the nucleus. Phosphorylated lamin A/C accumulates at regulatory regions of CAF effector genes, and expression of a Ser301 phosphomimetic mutant is sufficient to switch normal fibroblasts into tumor-promoting CAFs. These findings demonstrate that site-specific lamin phosphorylation can establish persistent stromal reprogramming within the TME [157].

Together, biochemical signaling pathways (e.g., AKT, ATM, caspases, and NF-κB) and physical cues (e.g., matrix rigidity and confinement) converge on lamin A/C to fine-tune nuclear stiffness, chromatin tethering, and the balance between deformability and robustness required for tumor cell invasion and survival. These findings support a model in which lamin A/C contributes to multiple cancer hallmarks through tied mechanical, structural, and signaling functions. Whether its role is tumor-suppressive or tumor-promoting depends on how lamin A/C expression levels and post-translational modifications position cells along a spectrum between nuclear deformability and mechanical robustness.

### 4.3. Expression Patterns and Prognostic Significance Across Tumors

The role of lamin A/C in cancer is significantly influenced by context, and its effects on tumor biology and clinical outcomes are still debated [107,132,135]. Lamin A/C expression varies among tumor types, influenced by tissue identity, differentiation state, mechanical environment, and simultaneous alterations in nuclear envelope components and signaling pathways. Moreover, lamin A and lamin C can show isoform-specific behaviors that are not always detected when the two are analyzed together [108,109,158]. This variability may help explain why lamin A/C can display tumor-suppressive, oncogenic, or dual behavior depending on cellular context.

In several epithelial and mesenchymal cancers, reduced lamin A/C expression correlates with poor clinical outcome, an observational pattern consistent with a tumor-suppressive role. In gastrointestinal cancers, for example, lamin A downregulation correlates with poor prognosis in gastric carcinoma [159] and with malignant progression in pancreatic intraductal papillary neoplasms [160]. Similarly, in pediatric tumors such as neuroblastoma, osteosarcoma, and Ewing sarcoma, low lamin A/C expression is associated with reduced patient survival, additionally, functional gain-of-function studies in cellular models suggest that lamin A/C re-expression reduces malignant phenotypes [111,126,131,161]. A similar situation is observed in lung cancer, where reduced lamin A/C correlates with nuclear atypia, metastatic dissemination, and poor outcome [138,162,163]. Additionally, in prostate cancer, diminished lamin A levels in low-grade tumors and increased expression in high-risk lesions indicate that A-type lamins may facilitate disease stratification. However, low levels of lamin A/C expression are associated with poor survival in aggressive cases, implying a complex, context-dependent role driven by disease stage, cellular state, and methodological discrepancies between studies [164,165,166].

On the other hand, in other cancers, such as glioblastoma, colorectal and ovarian carcinoma, elevated lamin A/C expression is associated with poorer survival [167,168,169]. In breast cancer, furthermore, multiple studies report an opposite relationship between lamin A/C expression and patient survival [103,126,170]. Additionally, comparative analyses of tumor and adjacent non-malignant tissue highlight higher lamin A/C levels in early-stage disease and favorable outcomes, and loss or heterogeneous expression of lamin A is common in high-grade tumors [171].

A different behavior was reported for liver cancer, where lamin A/C appears to have both oncogenic and tumor-suppressive functions. In hepatocellular carcinoma, high lamin A/C expression has been associated with increased proliferation, migration, and reduced survival in some studies [172], whereas *LMNA* downregulation may promote genomic instability during early tumorigenesis [107]. Studies in ovarian cancer have also provided conflicting results, with reports of both increased and decreased lamin A expression relative to normal tissue [108,137,173,174]. Nevertheless, higher lamin A levels are often associated with better prognosis and longer survival, whereas reduced lamin A correlates with nodal metastasis and adverse outcomes [108,137].

Table 1 recapitulates the aforementioned associated phenotypes and prognostic correlations with lamin A/C.

**Figure 5:**
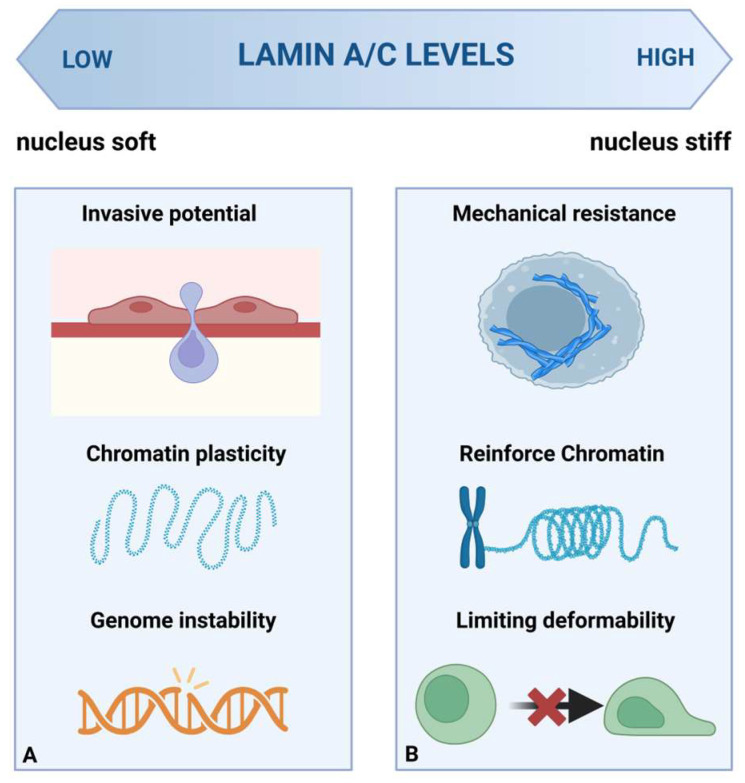
The impact of lamin A/C levels on nuclear mechanics, chromatin organization, and cell behavior. (**A**) Low lamin A/C expression is associated with a softer, more deformable nucleus that facilitates nuclear deformation during confined migration. This is linked to increased invasive potential. Reduced lamin A/C levels correlate with increased chromatin plasticity and susceptibility to genome instability, especially under mechanical stress. (**B**) High lamin A/C expression leads to a stiffer nucleus with greater mechanical resistance, stronger chromatin organization, and reduced nuclear deformability. Overall, lamin A/C levels act as key modulators of nuclear mechanics and genome organization, influencing cell migration and genomic stability in a context-dependent manner. Created in BioRender. Pasello, M. (2026) https://BioRender.com/fbp4wzq (accessed on 2 March 2026).

These observational cohorts and functional perturbation studies suggest that, rather than acting as a linear prognostic marker, lamin A/C functions as a context-dependent mechanical-epigenetic rheostat. The apparently contradictory observation that both low and high lamin A/C can correlate with poor prognosis can be rationalized within this framework by considering the dominant selective pressures operating across tumor types and disease stages. At low levels, reduced lamina stability may favor nuclear deformability, chromatin plasticity, and invasive potential, particularly where migration is set through confined spaces; however, this state may also increase nuclear fragility and genome instability (see Figure 5A). At higher levels, increased lamina stabilization may enhance resistance to mechanical stress, reinforce chromatin repression programs, or support therapy adaptation in some contexts, while potentially limiting extreme deformability (see Figure 5B). Tumors therefore occupy different positions along this axis depending on tissue stiffness, metastatic route, treatment-related stress, and oncogenic signaling programs. Interpretation is further complicated by isoform-specific effects, as selective loss of lamin A relative to lamin C is recurrent in metastatic disease and may differentially impact chromatin interactions and mechanotransduction. For example, in metastatic pleural adenocarcinoma, selective loss of lamin A, but not lamin C, has been reported, probably driven by microRNA regulation or alternative splicing [109]. Preferential loss of lamin A over lamin C is also observed in metastatic epithelial ovarian cancer [108]. This framework helps explain contrasting clinical observations and suggests that lamin A/C status should be interpreted in combination with nuclear mechanics, chromatin state, lamin isoform balance, and nuclear envelope composition.

## 5. Therapeutic Perspectives of Strategies Targeting Lamin A/C

Although a deeper understanding of lamin A/C functions in cancer is required to identify new therapeutic opportunities across tumor types, several emerging strategies that exploit pathways modulating *LMNA* expression, prelamin A processing, lamin A/C stability, or post-translational modifications have recently been explored. In particular, prelamin A maturation (e.g., ZMPSTE24 activity and farnesylation), post-translational modifications (such as Ser22 phosphorylation), lamin-interacting partners (including TPX2, ANKRD2, and ATM), and downstream mechanoresponsive pathways such as YAP/TAZ represent potentially exploitable vulnerabilities. Targeting these processes can increase nuclear stiffness, alter chromatin accessibility, and modulate DNA damage responses and transcriptional programs, thereby shifting-within specific contexts-the balance from invasion and plasticity toward differentiation, genome stability, and treatment sensitivity.

An important therapeutic approach includes the modulation of prelamin A maturation. Pharmacological inhibition of farnesyltransferase induces the accumulation of prelamin A and increases cellular susceptibility to DNA-damaging agents, such as reactive oxygen species (ROS), thereby limiting tumor aggressiveness [182]. Farnesyltransferase inhibitors promote the buildup of non-farnesylated prelamin A, a condition widely associated with aging-related processes including epigenetic alterations, inflammation, RNA activation, and altered mechanosignaling [183]. Accumulation of the lamin A precursor affects chromatin architecture and compromises the efficiency of the DNA damage response, making cells more vulnerable to genotoxic stress and less effective of neutralizing ROS [184]. In glioblastoma cells, prelamin A accumulation following treatment with SCH66336, a farnesyltransferase inhibitor, impairs the DNA damage response and, when combined with oxidative stress, reduces tumor aggressiveness and stem-like properties [182].

Similarly, inhibition of the zinc metalloprotease ZMPSTE24, which catalyzes the final step of lamin A maturation, prevents the conversion of prelamin A into mature lamin A, triggering cellular senescence and suppressing tumor cell migration [185,186]. Several ZMPSTE24 inhibitors induce intracellular prelamin A accumulation and inhibit cell migration in osteosarcoma, pancreatic adenocarcinoma, and colon carcinoma models. Among these, the analog 13a both induces prelamin A accumulation and inhibits migration, highlighting its potential as a lead compound. Importantly, 13a specifically targets ZMPSTE24, reducing off-target effects and supporting further preclinical development [187,188].

Beyond maturation, lamin A/C levels and stability are tightly regulated by interacting proteins that integrate mechanical stress, redox signaling, and cell-cycle control.

Reduction in ANKRD2 (ankyrin repeat domain-containing protein 2) has also been shown to significantly improve the efficacy of chemotherapeutic agents, suggesting a novel therapeutic strategy. In muscle cells, ROS exposure induces interaction between ANKRD2 and lamin A/C, regulating ANKRD2 nuclear shuttling [189,190]. ANKRD2 depletion selectively reduces lamin A expression at both transcript and protein levels without affecting lamin C and is accompanied by decreased expression of additional nuclear lamina components. Persistent DNA damage can further cause nuclear envelope rupture through lamin A/C degradation, indicating that reduced lamin A/C may arise either directly from ANKRD2 loss or indirectly from genome instability [191].

Importantly, depletion of nuclear lamina components sensitizes cells to replicative stress and chemotherapeutic agents. Cells lacking lamin A/C and displaying reduced cyclin D1 expression are particularly vulnerable to DNA crosslinking agents [104]. Consistently, in osteosarcoma models, ANKRD2 reduction significantly causes the cytotoxic efficacy of doxorubicin and cisplatin, supporting a combinatorial therapeutic strategy based on lamina destabilization [190].

Another critical lamin A/C regulator is TPX2 (targeting protein XkIp2), a microtubule-associated protein frequently overexpressed in cancer and associated with poor prognosis. TPX2 directly interacts with lamin A/C, modulating its expression and potentially stabilizing lamin A/C through Ser22 phosphorylation. In ovarian carcinoma, TPX2, an etiopathological factor associated with poor prognosis, promotes tumor growth by suppressing apoptosis and ROS production via modulation of lamin A/C stability, identifying TPX2 as a potential therapeutic target [149].

The DNA damage response kinases ATM (ataxia-telangiectasia mutated) and ATR (ATM and Rad3-related) play pivotal roles in maintaining lamin A/C levels and nuclear envelope integrity. Loss of ATM reduces lamin A levels across several cell types, resulting in more deformable nuclei and improved migration through confined environments. This suggests ATM as a key regulator linking genome stability, nuclear mechanics, and cell migration [144]. Similarly, the DNA damage response kinase ATR maintains nuclear envelope integrity by phosphorylating lamin A/C at Ser282 in response to DNA damage and replication stress. This modification regulates lamina assembly and chromatin–cytoskeleton attachment, and its disruption leads to lamin aggregation, G2-phase accumulation, and delayed mitotic entry, connecting mechanical stress responses to nuclear architecture and cell-cycle progression [191]. These observations suggest that therapeutic strategies targeting lamina assembly or nuclear envelope repair could be combined with DNA damage-based treatments to increase mechanical instability and impair adaptive responses.

Changes in the processing and stability of lamin A have a significant impact on downstream mechanotransduction pathways, particularly the YAP/TAZ signaling pathway.

Genetic or pharmacological induction of prelamin A accumulation restrains tumor cell motility and invasiveness, at least in part by suppressing the oncogenic YAP/TAZ signaling pathway, as demonstrated in Ewing sarcoma. Consistent with this mechanism, pharmacological inhibition of the YAP/TAZ-TEAD transcriptional complex using verteporfin or statins such as mevinolin, which inhibits the mevalonate pathway and farnesyl production [192], significantly reduces migration and metastatic burden in preclinical Ewing sarcoma models [131,193]. Notably, mevinolin has a dual antitumor effect by promoting prelamin A accumulation while simultaneously inhibiting mevalonate-dependent YAP/TAZ activity and inducing tumor cell differentiation [131]. Overall, these findings identify lamin A processing and downstream mechanotransduction pathways as actionable vulnerabilities in Ewing sarcoma and support therapeutic strategies aimed at restoring lamin A function while targeting YAP/TAZ signaling.

Small molecules that indirectly modulate lamin A/C expression further highlight its role in tumor progression. The phytoestrogen formononetin (FN) is a promising therapeutic compound with antiproliferative and anti-invasive properties supporting its potential as a single agent therapy [194]. Increasing evidence indicates potent antitumor effects of FN in breast cancer, ovarian cancer, and hepatocellular carcinoma [195,196]. In nasopharyngeal carcinoma, FN treatment induces dose-dependent downregulation of BCL-2, ERK1/2, lamin A/C, and cytokeratin-19, supporting a role for lamin modulation in suppressing tumor invasiveness [197].

Finally, CNOT1, a core component of the CCR4-NOT deadenylase complex, has emerged as an independent prognostic factor in osteosarcoma. CNOT1 directly interacts with lamin A, stabilizing the protein and promoting tumor progression through activation of Hedgehog signaling. Knockdown of CNOT1 significantly suppresses tumor growth in vitro and in vivo, while *LMNA* overexpression rescues this effect, confirming the functional relevance of this interaction. These results suggest that the CNOT1-LMNA-Hedgehog pathway could be a possible treatment target for osteosarcoma [198].

Table 2 recapitulates the aforementioned therapeutic strategies targeting lamin A/C.

## 6. Conclusions and Future Perspectives

Lamin A/C has been proven to be a key mechano-epigenetic link between nuclear structure and gene regulation, helping to organize 3D genome and the balance between chromatin flexibility and stability. Mechanical and biochemical cues, through changes in lamin A/C levels, isoforms, processing, and post-translational modifications, can leave lasting effects on nuclear mechanics and transcriptional programs. In cancer, these same mechanisms act in a strongly context-dependent way, affecting proliferation, migration, survival under stress, and therapy response, with very mixed prognostic patterns. Rather than a simple oncogene or tumor suppressor, lamin A/C sets a functional “operating range” that influences trade-offs between nuclear deformability, genomic stability, and adaptability. Early studies suggest this range is pharmacologically tunable through interventions on lamin A/C abundance/stability, prelamin A maturation, lamin A/C phosphorylation, upstream regulators, and lamin A/C-linked signaling axes, and lamin A/C features may help stratify patients.

Within this emerging framework, several conceptual challenges may shape the next phase of research:

1. Systematic, high-resolution mapping of lamin A/C expression, isoform composition, processing intermediates, post-translational modifications, and subnuclear localization, integrated with single-cell, spatial, and mechanical analysis, could reveal whether distinct lamin A/C configurations correlate with differentiation, tissue stiffness, or disease trajectory.
2. It remains unclear in many settings whether changes in lamin A/C actively drive oncogenic programs, merely permit them, or arise as downstream adaptations to altered mechanical or metabolic states. Many reported associations between lamin A/C perturbations and signaling pathways, including YAP/TAZ, EMT programs, DNA damage responses, and stemness networks, remain correlative. Mechanistic studies in model systems that incorporate both tumor and stromal components, including cancer-associated fibroblasts, will be essential to distinguish driver from bystander effects and to determine how lamin A/C-dependent chromatin organization interfaces with specific oncogenic and drug-response circuits.
3. Translating lamin A/C biology into clinically meaningful strategies will require careful calibration. While proof-of-concept studies suggest that prelamin A processing, *LMNA* stability, or lamin A/C-sensitive signaling pathways might be therapeutically exploitable, key questions remain regarding specificity, safety, and therapeutic windows. Given the essential roles of lamin A/C in normal, mechanically stressed tissues, future strategies may need to emphasize context-dependent modulation rather than extensive inhibition, potentially guided by lamin A/C-based biomarkers.
4. Tumors may be stratified and targeted through vulnerabilities linked to lamin A/C. Lamin A/C features could define states of nuclear mechanics and chromatin plasticity that predict sensitivity to DNA/chromatin-directed therapies. In particular, low lamin A/C, while enabling deformability and migration, may impose liabilities under mechanical stress, such as nuclear rupture and genome instability, which can be therapeutically exploited. This suggests the need for partial, transient, and combinatorial approaches that maximize tumor selectivity and limit toxicity.
5. Beyond its well-established functions in nuclear mechanics and genome organization, lamin A/C has been linked to additional processes relevant to cancer. Recent studies suggest that altered lamin A/C expression may influence tumor-immune interactions [199], potentially contributing to immune evasion by affecting nuclear architecture and inflammatory signaling [200]. Lamin-low conditions have also been associated with nuclear rupture, micronucleus formation, and chromothripsis, which provides a mechanistic link between nuclear fragility and genome rearrangements [37]. Additionally, emerging evidence [201] points to a role for lamin A/C in metabolic regulation, including lipid and glucose homeostasis, which may intersect with oncogenic metabolic reprogramming. Finally, age-related changes in lamin A/C expression and post-translational modification suggest that lamin dysfunction may contribute to the increased cancer risk associated with aging [202,203]. Although these fields remain largely unexplored, they suggest important directions for future investigation.

Altogether, lamin A/C can be viewed as both a potential readout and a modulator of the mechano-epigenetic state of tumor and stromal cells. Understanding how this network is reshaped across cancer types, and whether it can be selectively tuned without compromising normal tissue function, may ultimately determine whether nuclear lamina biology can be translated into robust biomarkers and actionable therapeutic vulnerabilities.

## Figures and Tables

**Table 1:** Lamin A/C expression patterns and associated phenotypes across human cancers.

Tumor Type	Lamin A/C Expression	Level of Analysis	Main Associated Phenotype	Clinical Relevance	Proposed Functional Role	Refs.
Ovarian serous cancer	Increased	Protein; mRNA	Genomic stability, apoptotic evasion, resistance to etoposide	Higher-stage tumors	Oncogenic	[169,175]
Colorectal cancer	Increased	Protein (IHC)	Increased cell motility	Reduced overall survival	Oncogenic	[168]
Glioblastoma	Increased	Protein; gene (*LMNA)*	Increased aggressiveness and tumorigenicity	Poor prognosis	Oncogenic	[167]
Prostate cancer	Increased/Reduced	Protein (IHC, WB)	Increased proliferation, migration, invasion; EMT activation	High-risk disease; increased lymph node metastasis	Context-dependent	[136,165,176]
Epithelial ovarian cancer	Reduced	Protein (IHC)	Increased metastasis; reduced survival	Poor prognosis	Tumor-suppressive	[108]
Colon cancer (stage II–III)	Reduced	Protein (IHC)	Increased recurrence risk	Unfavorable outcome	Tumor-suppressive	[177]
Breast cancer	Reduced	Protein; mRNA	Enhanced invasion, proliferation; nuclear deformability	Adverse clinical outcome	Tumor-suppressive	[103,139,170]
Gastric cancer	Reduced	Protein (IHC)	Poor differentiation	Poor patient outcome	Tumor-suppressive	[159]
Non–small cell lung cancer	Reduced	Protein; mRNA	EMT activation; erlotinib resistance	Therapy resistance	Tumor-suppressive	[178]
Cervical cancer	Reduced	Protein (IHC)	Increased cancer susceptibility	Risk factor for disease development	Tumor-suppressive	[179]
Leukemia	Reduced	Gene expression	Altered nuclear architecture	Disease aggressiveness	Tumor-suppressive	[180,181]
Lymphoma	Reduced	Gene expression	Epigenetic silencing of *LMNA*	Poor survival	Tumor-suppressive	[180,181]
Ewing sarcoma	Reduced	Protein; functional assays	Increased migration, invasiveness, metastasis	Reduced overall survival	Tumor-suppressive	[131]
Osteosarcoma	Reduced	Protein; mechanobiology assays	Increased proliferation and migration	Reduced overall survival	Tumor-suppressive	[125,126,127]

**Table 2:** Therapeutic strategies targeting lamin A/C-associated pathways in cancer.

Strategies	Mechanism of Action	Effect on Lamin A/C	Effects on Cancer	Cancer Type	Refs.
Farnesyltransferase inhibitors	Inhibition of prelamin A farnesylation	Accumulation of prelamin A	Chromatin disorganization. Impaired DNA damage response. Increased ROS sensitivity.	Glioblastoma	[182]
ZMPSTE24 inhibitors	Block final maturation step of lamin A	Persistent prelamin A accumulation	Cellular senescence. Reduced migration and invasiveness.	Osteosarcoma. Pancreatic Adenocarcinoma. Colon carcinoma	[187,188]
ANKRD2 depletion	Loss of lamin interacting redox/mechanical regulator	Selective reduction in lamin A (not lamin C)	Enhanced sensitivity to DNA damage and chemotherapy. Nuclear envelope fragility.	Osteosarcoma	[190]
TPX2 inhibition	Modulation of lamin A/C expression	Modulation of lamin A/C stability	Increased apoptosis and ROS. Reduced tumor growth.	Ovarian carcinoma	[149]
ATM loss/inhibition	Impaired DNA damage signaling	Reduced lamin A levels	Enhanced migration through confined spaces. Genome instability.	Multiple tumor types	[144]
ATR signaling disruption	Loss of lamin A/C Ser282 phosphorylation	Defective lamina assembly. Lamin aggregation	G2 arrest. Delayed mitotic entry. Nuclear envelope instability.	Homologous-recombination-deficient (HRD) cells	[191]
YAP/TAZ-TEAD inhibition	Restore lamin A functions	Prelamin A accumulation	Reduced migration and metastasis. Induction of differentiation	Ewing sarcoma	[131,193]
Formononetin	Downregulation of oncogenic and cytoskeletal programs	Reduced lamin A/C expression	Decreased proliferation and invasion	Nasopharyngeal carcinoma. Breast carcinoma. Ovarian carcinoma. HCC	[195,196,197]
CNOT1 (CCR4-NOT complex)	Stabilization of lamin A and activation of Hedgehog signaling	Increased lamin A stability	Tumor growth and progression	Osteosarcoma	[198]

## Data Availability

No new data were created or analyzed in this study. Data sharing is not applicable to this article.
